# Long-Term Visit-to-Visit Mean Arterial Pressure Variability and the Risk of Heart Failure and All-Cause Mortality

**DOI:** 10.3389/fcvm.2021.665117

**Published:** 2021-06-04

**Authors:** Menghui Liu, Xiaohong Chen, Shaozhao Zhang, Yifen Lin, Zhenyu Xiong, Xiangbin Zhong, Yue Guo, Xiuting Sun, Huimin Zhou, Xingfeng Xu, Lichun Wang, Xinxue Liao, Xiaodong Zhuang

**Affiliations:** ^1^Department of Cardiology, The First Affiliated Hospital of Sun Yat-Sen University, Guangzhou, China; ^2^Key Laboratory on Assisted Circulation, Ministry of Health, Guangzhou, China; ^3^Department of Otorhinolaryngology, The Third Affiliated Hospital of Sun Yat-Sen University, Guangzhou, China

**Keywords:** blood pressure variability, variability independent of the mean, mean arterial pressure, heart failure, all-cause mortality

## Abstract

**Background:** Systolic or diastolic blood pressure (BP) variability is associated with an increased risk of cardiovascular events. We assessed whether BP variability measured by mean arterial pressure (MAP) was associated with increased risk of heart failure (HF) and death in individuals with or without hypertension.

**Methods:** We evaluated 9,305 Atherosclerosis Risk in Communities (ARIC) study participants with or without hypertension and calculated BP variability based on MAP values from visit 1 to 4 [expressed as standard deviation (SD), average real variability (ARV), coefficient of variation (CV), and variability independent of the mean (VIM)]. Multivariate-adjusted Cox regression model and restricted cubic spline curve were used to evaluate the associations of MAP variability with all-cause mortality and HF.

**Results:** During a median follow-up of 16.8 years, 1,511 had an HF event and 2,903 died. Individuals in the highest quartile of VIM were both associated with a 21% higher risk of all-cause mortality [hazard ratio (HR), 1.21; 95% CI, 1.09–1.35] and HF (HR, 1.21; 95% CI, 1.04–1.39) compared with the lowest quartile of VIM. Cubic spline curves reveal that the risk of deaths and HF increased with MAP variability when it reached a higher level. Results were similar in individuals with normotension (all-cause mortality: HR, 1.30; 95% CI, 1.09–1.55; HF, HR, 1.49; 95% CI, 1.12–1.98).

**Conclusions:** In individuals with or without hypertension, greater visit-to-visit MAP variability was associated with a higher risk of all-cause mortality and HF, indicating that the BP variability assessed by MAP might be a potential risk factor for HF and death.

## Introduction

The 2017 American College of Cardiology (ACC)/American Heart Association (AHA) (1) and 2018 European Society of Cardiology (ESC)/European Society of Hypertension (ESH) (2) blood pressure (BP) guideline recommends using a single measurement or the average of BP levels assessed over time to screen for and manage high BP in adults, while occasional BP increase or reduction has not been taken seriously by clinicians. Although fluctuation of BP is physiological (3), a growing number of clinical and observational studies have demonstrated that elevated BP variability contributes to the risk of cardiovascular disease (CVD) and death, independently of mean BP (4–12). Thus, BP variability has increasingly been recognized as a novel CVD risk factor that can provide more accurate estimates for the clinical outcomes in adults (13).

Previous studies exploring the independent risk of BP variability tended to focus on systolic BP (SBP) (4–10, 14), diastolic BP (DBP) (6, 9), or pulse pressure (PP) (11, 12). However, few studies to date have evaluated the potential impact of long-term mean arterial pressure (MAP) variability on CVD and death. MAP is considered to be a steady component along which BP fluctuates between the SBP and DBP levels and a main driving force for vital organ perfusion (15). The clinical prognostic power of MAP in predicting the risk for CVD was reported to be even superior to that of SBP and DBP (16). Moreover, several studies showed that MAP was associated with a hospital or long-term mortality in patients with cardiogenic shock or heart failure (HF) (17, 18). Therefore, to provide evidence on BP variability assessed by MAP, our study was to evaluate the association of long-term visit-to-visit MAP variability with the risks of HF and death in community population using the Atherosclerosis Risk in Communities (ARIC) study (19).

## Materials and Methods

### Study Design and Study Population

The ARIC study is an ongoing, community-based, prospective cohort study designed to assess the risk factors for CVD. A total of 15,792 participants between 45 and 64 years were recruited between 1987 and 1989 from four US population centers: Forsyth County, North Carolina; Jackson, Mississippi; Washington County, Maryland; and Northwestern suburbs of Minneapolis, Minnesota. The participants were examined initially every 3 years to conduct the three subsequent visits, with the second examination in 1990–1992 (visit 2), the third in 1993–1995 (visit 3), and the fourth in 1996–1998 (visit 4). Fifteen years later, a fifth visit occurred between 2011 and 2013. Details of the study design have been published elsewhere (19).

For the present study, the 9,305 participants were included for analyses, excluding those missing data in the public access data sets (*n* = 809), those who did not attend visit 4 (*n* = 4,013), those missing mean MAP data (*n* = 499) or information on covariates (*n* = 527), and those whose HF occurred before or at visit 4 (*n* = 639) (Supplementary Figure 1). The ARIC study was approved by the institutional review boards at all participating institutions, and informed consent of all participants was obtained in writing at each examination.

### Visit-to-Visit MAP Variability

Three seated BP readings from participants sitting in a quiet room for 5 min were obtained by technicians utilizing random-zero sphygmomanometers. The average of the last two measures was used for analysis. MAP has been calculated by BP cuff measurements using a traditional formula, which states that the MAP equals 1/3 × SBP plus 2/3 × DBP. To improve the robustness, the MAP_2_ calculated by another formula (MAP_2_ = DBP + 0.412 × PP) (20) was also used in the sensitivity analysis. Mean MAP levels were calculated across four visits (visit 1, 2, 3, and 4) for each participant.

The long-term visit-to-visit MAP variability measurements included standard deviation (SD), average real variability (ARV), coefficient of variation (CV), and variability independent of the mean (VIM). The formulas of each MAP variability are shown in the Supplementary Figure 2 (21, 22).

After calculating each MAP variability, we conducted the Pearson's correlation among mean MAP and them (Supplementary Table 1). The SD, CV, and ARV of MAP were correlated with mean MAP (Pearson *r* = 0.12–0.33), but the VIM of MAP was poorly correlated with mean MAP (Pearson *r* = −0.052) and had a strong correlation with SD, CV, and ARV (Pearson *r* = 0.78–0.98). Thus, to distinguish the impact of MAP variability from that of mean MAP on outcomes, the VIM was used to measure visit-to-visit MAP variability in the primary analysis. The SD, CV, and ARV of MAP were just used in the secondary analyses.

### Outcome Ascertainment

The ascertainment of deaths and classification of HF, coronary heart disease (CHD), and stroke events in ARIC has been described previously (23–26). All-cause mortality was defined as death from any cause and ascertained through the review of death certificates and hospital discharge records, supplemented by physician questionnaires for out-of-hospital deaths or informant interviews (23). Prior to 2005, ARIC did not collect record material other than discharge codes for incident HF hospitalizations. Therefore, we defined incident hospitalized HF by diagnostic code [International Classification of Diseases, Ninth Revision (ICD-9) code 428] from hospital discharges until 2004 (24). After 2005, ARIC staff members abstracted a broad range of hospital records for potential HF events to ascertain HF hospitalization (23). CVD was defined as the first occurrence of CHD or stroke after visit 4. CHD events were adjudicated by an ARIC end points committee and included fatal CHD, definite or probable myocardial infarction (MI), and silent MI (25). The physician reviewers obtained hospital records for possible stroke-related hospitalizations and collected information on fatal stroke through linkage with the National Death Index. Definite or probable stroke events were identified by a computer algorithm and adjudicated by physician reviewers (26).

### Statistical Analyses

Baseline characteristics at visit 4 are presented as the mean (SD) for continuous variables or number (%) for categorical variables. After calculating each MAP variability, the population was categorized into four groups by quartile value. We constructed Kaplan–Meier graphs and used the log-rank test to assess differences in the risks of all-cause mortality and HF among four groups. The multivariable-adjusted Cox proportional hazards models were used to estimate the hazard ratio (HR) (95% CIs) for incident death, HF, and CVD associated with the higher MAP variability, including the following covariates: model 1—age, sex, race at visit 4; model 2—variables in model 1 plus body mass index (BMI), education level, smoking status, drinking status, total cholesterol, low-density lipoprotein cholesterol (LDL-C), high-density lipoprotein cholesterol (HDL-C), triglyceride, fasting glucose, estimated glomerular filtration rate (eGFR), prevalent hypertension, diabetes mellitus, CHD, MI, and stroke, antihypertensive medicine, aspirin, statin at visit 4; and model 3—variables in model 2 and SBP, DBP at visit 4, and mean of MAP from visit 1 to 4. We also analyzed the effect of MAP variability on the subsequent death and HF as a continuous variable for a restricted cubic spline with three knots and presented it graphically along with the best-fitted straight line. The primary analysis was based on the MAP variability measured by VIM.

The subgroup analyses of key variables (age, sex, race, BMI, LDL-C, eGFR, hypertension, diabetes, and smoking status) were also performed to compare the risks of all-cause mortality and HF between the highest (VIM Q4) and the other three quartiles (VIM Q1 + Q2 + Q3) of MAP variability. An interaction term between key variable and MAP variability was individually added to the adjusted Cox model 3, and the *P*-values and CIs for these associations were estimated. The sensitivity analyses were conducted by MAP variability measured by SD, CV, and ARV. Considering the 44.9% of participants with hypertension, to avoid the impact of hypertension on the association between MAP variability and outcomes, we also conducted a sensitivity analysis in participants with normotension (*n* = 4,600). Those with hypertension (*n* = 4,178) or taking antihypertensive (*n* = 527) were excluded. In addition, the associations between MAP_2_ variability and outcomes were further assessed using multivariable-adjusted Cox proportional hazards models in the sensitivity analysis. All the tests were two-sided with *P* < 0.05 considered significant. Statistical analyses were conducted using the Stata Version 14 (StataCorp, College Station, TX, USA) and the R language (version 3.5.0.12).

## Results

Population characteristics are shown in Table 1. Of the 9,305 participants from the ARIC study included in the current analysis with an average of 62.8 years old at visit 4; 44.9% were male and 18.8% were black. Compared with the lower VIM of MAP (Q1–Q3), the participants with the highest quartile of VIM (Q4) were older, more likely female, less likely white, more likely to have a comorbidity (hypertension, diabetes, CHD, stroke) and had lower levels of eGFR.

**Table 1:** Characteristics of each group categorized by the VIM of MAP at visit 4.

**Characteristics**	**Total**	**VIM Q1**	**VIM Q2**	**VIM Q3**	**VIM Q4**	***P*-value**
No.	9,305	2,326	2,327	2,326	2,326	
Age, years	62.8 (5.7)	62.4 (5.5)	62.9 (5.6)	62.9 (5.7)	63.2 (5.7)	<0.001
Sex, no. (%)						<0.001
Men	4,178 (44.9)	1,174 (49.5)	1,077 (46.3)	982 (42.2)	945 (40.6)	
Women	5,127 (55.1)	1,152 (50.5)	1,250 (53.7)	1,344 (57.8)	1,381 (59.4)	
Race, no. (%)						<0.001
Black	1,753 (18.8)	357 (15.3)	412 (17.7)	460 (19.8)	524 (22.5)	
White	7,552 (81.2)	1,969 (84.7)	1,915 (82.3)	1,866 (80.2)	1,802 (77.5)	
BMI, kg/m^2^	28.5 (5.4)	28.5 (5.0)	28.6 (5.4)	28.5 (5.5)	28.6 (5.6)	0.808
Systolic BP, mm Hg	126.9 (18.6)	125.8 (16.5)	126.4 (17.3)	127.1 (18.5)	128.4 (21.8)	0.012
Diastolic BP, mm Hg	70.8 (10.1)	71.2 (8.4)	71.3 (9.4)	70.7 (10.3)	70.0 (12.0)	<0.001
MAP, mm Hg	89.5 (11.4)	89.4 (9.4)	89.7 (10.5)	89.5 (11.4)	89.4 (13.7)	0.686
Total cholesterol, mmol/L	5.2 (0.9)	5.2 (0.9)	5.2 (0.9)	5.2 (1.0)	5.2 (1.0)	0.816
HDL-C, mmol/L	1.3 (0.4)	1.3 (0.4)	1.3 (0.4)	1.3 (0.4)	1.3 (0.4)	0.008
LDL-C, mmol/L	3.2 (0.9)	3.2 (0.8)	3.2 (0.8)	3.2 (0.9)	3.2 (0.9)	0.983
Triglyceride, mmol/L	1.6 (0.8)	1.6 (0.8)	1.5 (0.8)	1.5 (0.7)	1.6 (0.8)	0.338
Fasting glucose, mmol/L	6.1 (2.0)	6.1 (2.0)	6.0 (1.9)	6.0 (2.0)	6.1 (2.0)	0.178
eGFR, ml/min/1.73 m^2^	86.1 (15.6)	86.6 (14.2)	86.8 (14.6)	86.1 (15.5)	84.8 (17.7)	0.018
Diabetes mellitus, no. (%)	1,378 (14.8)	345 (14.8)	293 (12.6)	341 (14.7)	399 (17.2)	<0.001
Hypertension, no. (%)	4,178 (44.9)	832 (35.8)	951 (40.9)	1,066 (45.8)	1,329 (57.1)	<0.001
Coronary heart disease, no. (%)	671 (7.2)	131 (5.6)	144 (6.2)	167 (7.2)	229 (9.8)	<0.001
Myocardial infarction, no. (%)	556 (6.0)	112 (4.8)	120 (5.2)	140 (6.0)	184 (7.9)	<0.001
Stroke, no. (%)	182 (2.0)	28 (1.2)	34 (1.5)	44 (1.9)	76 (3.3)	<0.001
Education level, no. (%)						<0.001
Basic or 0 year	1,610 (17.3)	331 (14.2)	420 (18.1)	401 (17.2)	458 (19.7)	
Intermediate	3,959 (42.5)	1,020 (43.9)	952 (40.9)	100.8 (43.3)	979 (42.1)	
Advanced	3,736 (40.2)	975 (41.9)	955 (41.0)	917 (39.4)	889 (38.2)	
Smoking, no. (%)						<0.001
Current smoker	1,333 (14.3)	257 (11.0)	315 (13.5)	336 (14.4)	425 (18.3)	
Former smoker	4,081 (43.9)	1,065 (54.8)	1,019 (43.8)	979 (42.1)	1,018 (43.9)	
Never smoker	3,891 (41.8)	1,004 (43.2)	993 (42.7)	1,011 (43.5)	883 (38.0)	
Drinking, no. (%)						<0.001
Current drinker	4,781 (51.4)	1,291 (55.5)	1,228 (52.8)	1,172 (50.4)	1,090 (46.9)	
Former drinker	2,684 (28.8)	600 (25.8)	645 (27.7)	669 (28.8)	770 (33.1)	
Never drinker	1,840 (19.8)	435 (18.7)	454 (19.5)	485 (20.9)	466 (20.0)	
Aspirin, no. (%)	5,227 (56.2)	1,269 (54.6)	1,331 (57.2)	1,298 (55.8)	1,329 (57.1)	0.215
Statin, no. (%)	1,010 (10.9)	213 (9.2)	229 (9.8)	254 (10.9)	314 (13.5)	<0.001
Antihypertensive, no. (%)	3,751 (40.3)	755 (32.5)	847 (36.4)	950 (40.8)	1,199 (51.5)	<0.001
VIM	6.2 (3.2)	2.8 (0.8)	4.8 (0.5)	6.7 (0.6)	10.6 (2.6)	<0.001

*Continuous variables are presented as mean (SD), and categorical variables are presented as percentage*.

*MAP, mean arterial pressure; VIM, variability independent of the mean; BMI, body mass index; BP, blood pressure; HDL-C, high-density lipoprotein cholesterol; LDL-C, low-density lipoprotein cholesterol; eGFR, estimated glomerular filtration rate*.

### Association of MAP Variability Measured by Vim With Outcomes

**Figure 1:**
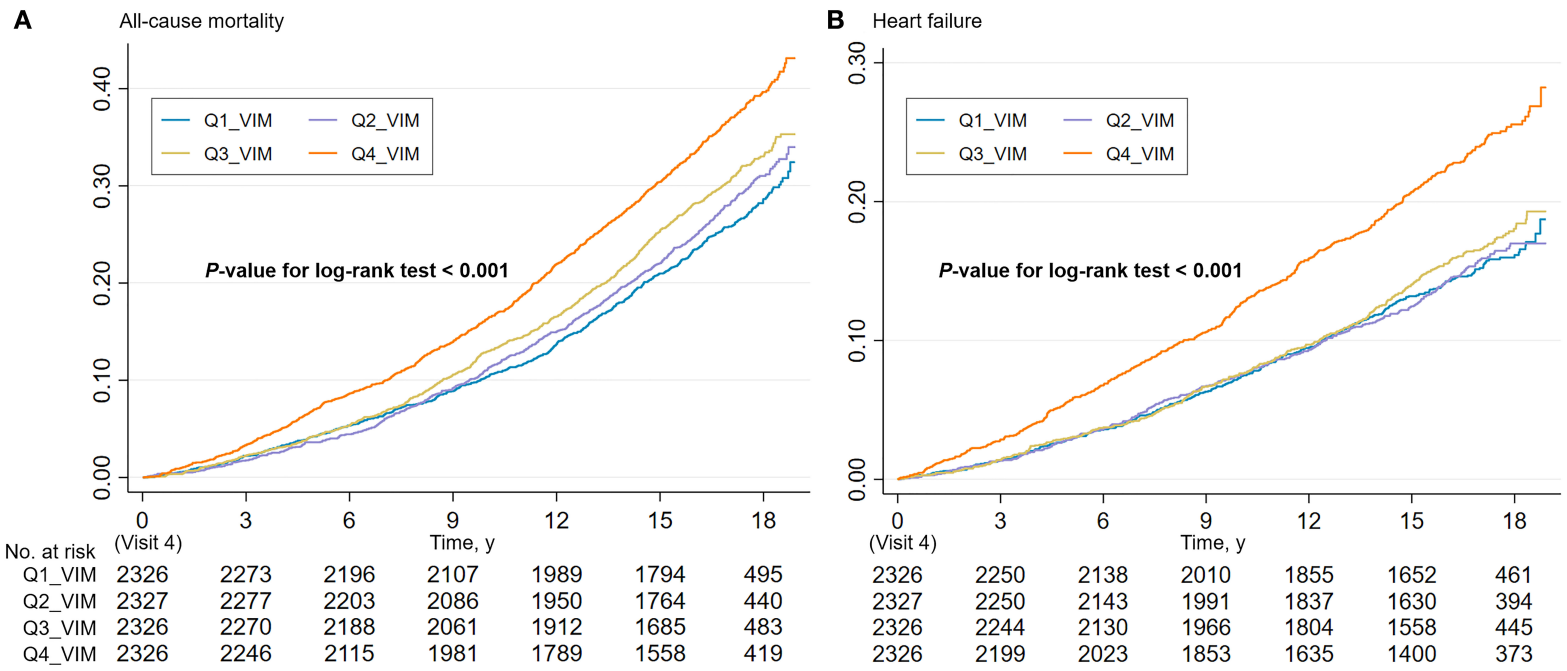
Cumulative incidence estimates (Kaplan–Meier) for the **(A)** all-cause mortality and **(B)** heart failure in four groups by quartile value of MAP variability (VIM). MAP, mean arterial pressure; VIM, variability independent of the mean.

**Figure 2:**
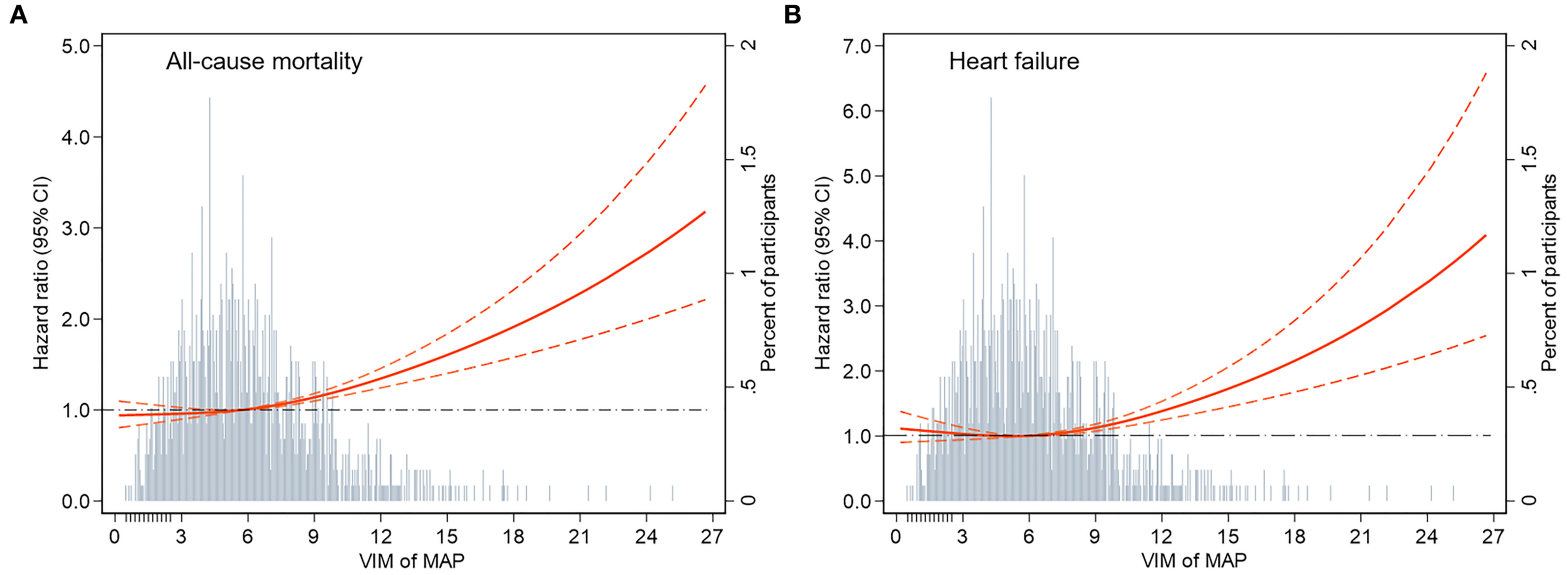
Adjusted hazard ratios (95% CI) for the association of MAP variability measured by VIM with incident **(A)** all-cause mortality and **(B)** heart failure. Hazard ratios (indicated by a red solid line) and 95% CIs (red dotted lines) are derived from Cox proportional hazard regression models adjusted for age, sex, race, BMI, education level, smoking status, drinking status, total cholesterol, LDL-C, HDL-C, triglyceride, fasting glucose, eGFR, prevalent hypertension, diabetes mellitus, coronary heart disease, myocardial infarction, stroke, antihypertensive medicine, aspirin, statin, SBP, DBP at visit 4, and mean of MAP from visit 1 to 4. The VIM of MAP was centered at the sample median and modeled using a restricted cubic spline with knots at the 5th, 50th, and 95th percentiles. The black dotted line is the reference line as hazard ratio = 1. Histograms represent the frequency distribution of MAP variability (VIM). MAP, mean arterial pressure; VIM, variability independent of the mean; BMI, body mass index; LDL-C, low-density lipoprotein cholesterol; HDL-C, high-density lipoprotein cholesterol; eGFR, estimated glomerular filtration rate; SBP, systolic blood pressure; DBP, diastolic blood pressure.

During a median follow-up of 16.8 years, 2,903 all-cause deaths and 1,511 HF events occurred. The Kaplan–Meier survival function curves showed higher risks of incident death and HF in participants with the highest quartile of VIM compared with the other three quartiles (VIM Q1–Q3) (Figure 1). In the multivariable-adjusted model, the highest quartile of VIM was both associated with a 21% higher risk of all-cause mortality (HR, 1.21; 95% CI, 1.09–1.35) and HF (HR, 1.21; 95% CI, 1.04–1.39) compared with the lowest quartile of VIM (Q1) (Table 2). No significant differences in the risk of incident death and HF were found in moderate quartile of VIM (Q2) (all-cause death: HR, 1.01; 95% CI, 0.91–1.13; HF: HR, 0.93; 95% CI, 0.80–1.09) and in high quartile of VIM (Q3) (all-cause death: HR, 1.08; 95% CI, 0.97–1.21; HF: HR, 0.95; 95% CI, 0.81–1.10) (Table 2). Cubic spline curves between MAP variability measured by VIM and the HR of incident death and HF are presented in Figure 2 and reveal that the risk of deaths and HF increased with MAP variability when it reached a higher level. In addition, a similar association between MAP variability and incident CVD was observed in Model 1 (*P* for trend = 0.006) but became non-significant after adjustment for traditional risk factors (all *P* > 0.05; Supplementary Table 2).

**Table 2:** Association of visit-to-visit MAP variability measured by VIM with all-cause mortality and heart failure events.

**Variability**	**Model 1**		**Model 2**		**Model 3**	
	**Hazard ratio (95% CI)**	***P*-value**	**Hazard ratio (95% CI)**	***P*-value**	**Hazard ratio (95% CI)**	***P*-value**
**All-cause mortality**						
VIM Q1	1 (ref.)	–	1 (ref.)	–	1 (ref.)	–
VIM Q2	1.05 (0.94–1.17)	0.409	1.00 (0.90–1.12)	0.992	1.01 (0.91–1.13)	0.820
VIM Q3	1.16 (1.05–1.30)	0.005	1.07 (0.96–1.19)	0.212	1.08 (0.97–1.21)	0.150
VIM Q4	1.46 (1.32–1.62)	<0.001	1.20 (1.08–1.34)	0.001	1.21 (1.09–1.35)	<0.001
*P* for trend	<0.001		<0.001		<0.001	
**Heart failure**						
VIM Q1	1 (ref.)	–	1 (ref.)	–	1 (ref.)	–
VIM Q2	0.97 (0.83–1.13)	0.658	0.91 (0.78–1.06)	0.228	0.93 (0.80–1.09)	0.377
VIM Q3	1.04 (0.90–1.21)	0.606	0.93 (0.80–1.08)	0.331	0.95 (0.81–1.10)	0.462
VIM Q4	1.56 (1.35–1.79)	<0.001	1.18 (1.03–1.37)	0.022	1.21 (1.04–1.39)	0.011
*P* for trend	<0.001		0.010		0.006	

*Model 1: adjusted for age, sex, race at visit 4; Model 2: adjusted for Model 1 + education level, BMI, smoking status, drinking status, total cholesterol, HDL-C, LDL-C, triglyceride, fasting glucose, eGFR, prevalent hypertension, diabetes mellitus, coronary heart disease, myocardial infarction, stroke, antihypertensive medicine, aspirin, statin at visit 4; Model 3: adjusted for Model 2 + SBP, DBP at visit 4 and mean of MAP from visit 1 to 4*.

*MAP, mean arterial pressure; VIM, variability independent of the mean; BMI, body mass index; HDL-C, high-density lipoprotein cholesterol; LDL-C, low-density lipoprotein cholesterol; eGFR, estimated glomerular filtration rate; SBP, systolic blood pressure; DBP, diastolic blood pressure*.

**Figure 3:**
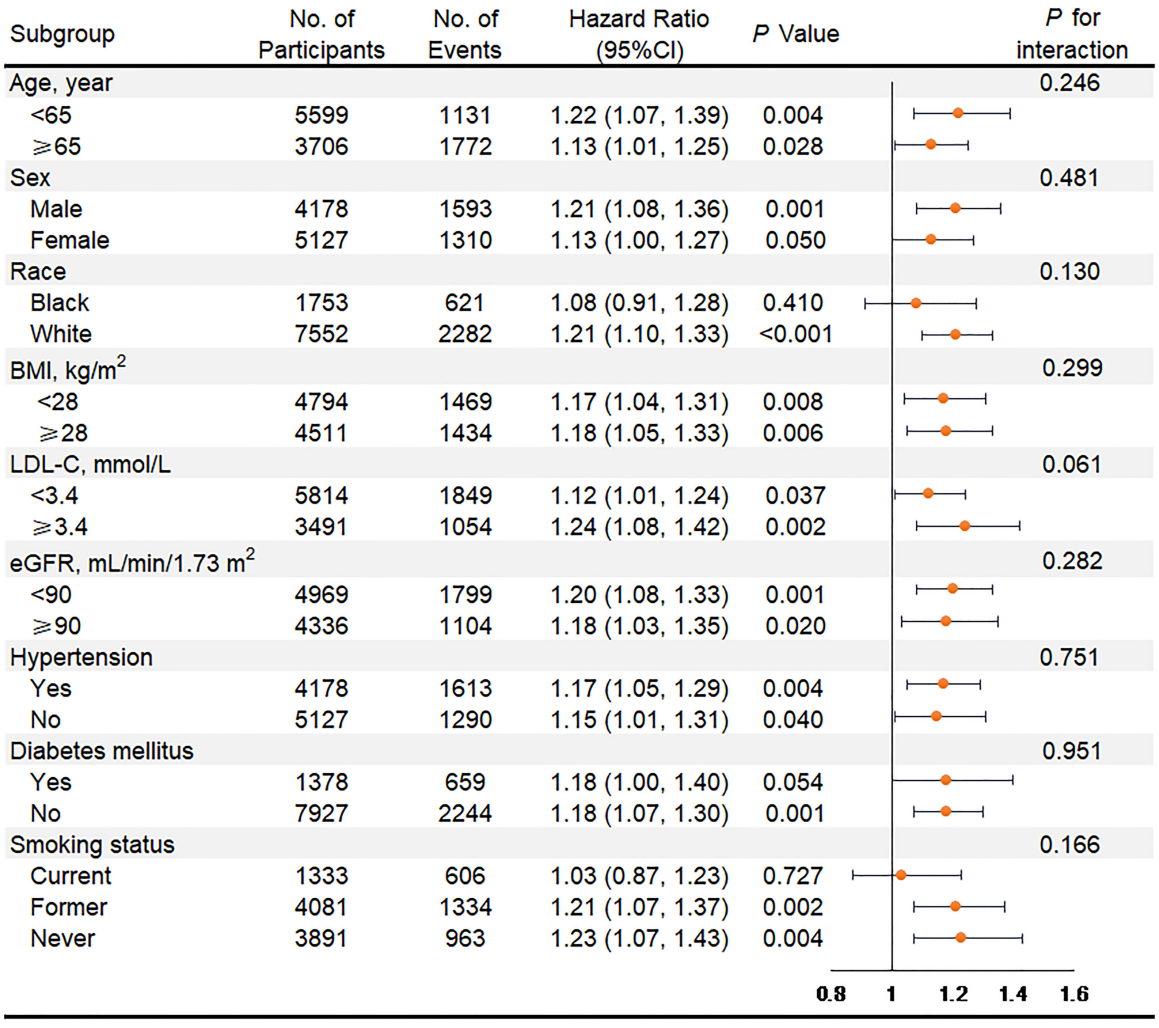
Association of the highest quartile of MAP variability group (VIM Q4) compared with the lower MAP variability group (VIM Q1 + Q2 + Q3) for all-cause mortality in key subgroups. Hazard ratios and 95% CIs were obtained after individually removing the original variable from the Cox Model 3 that adjusted for age, sex, race, BMI, education level, smoking status, drinking status, total cholesterol, LDL-C, HDL-C, triglyceride, fasting glucose, eGFR, prevalent hypertension, diabetes mellitus, coronary heart disease, myocardial infarction, stroke, antihypertensive medicine, aspirin, statin, SBP, DBP at visit 4, and mean of MAP from visit 1 to 4. MAP, mean arterial pressure; VIM, variability independent of the mean; BMI, body mass index; LDL-C, low-density lipoprotein cholesterol; HDL-C, high-density lipoprotein cholesterol; eGFR, estimated glomerular filtration rate; SBP, systolic blood pressure; DBP, diastolic blood pressure.

**Figure 4:**
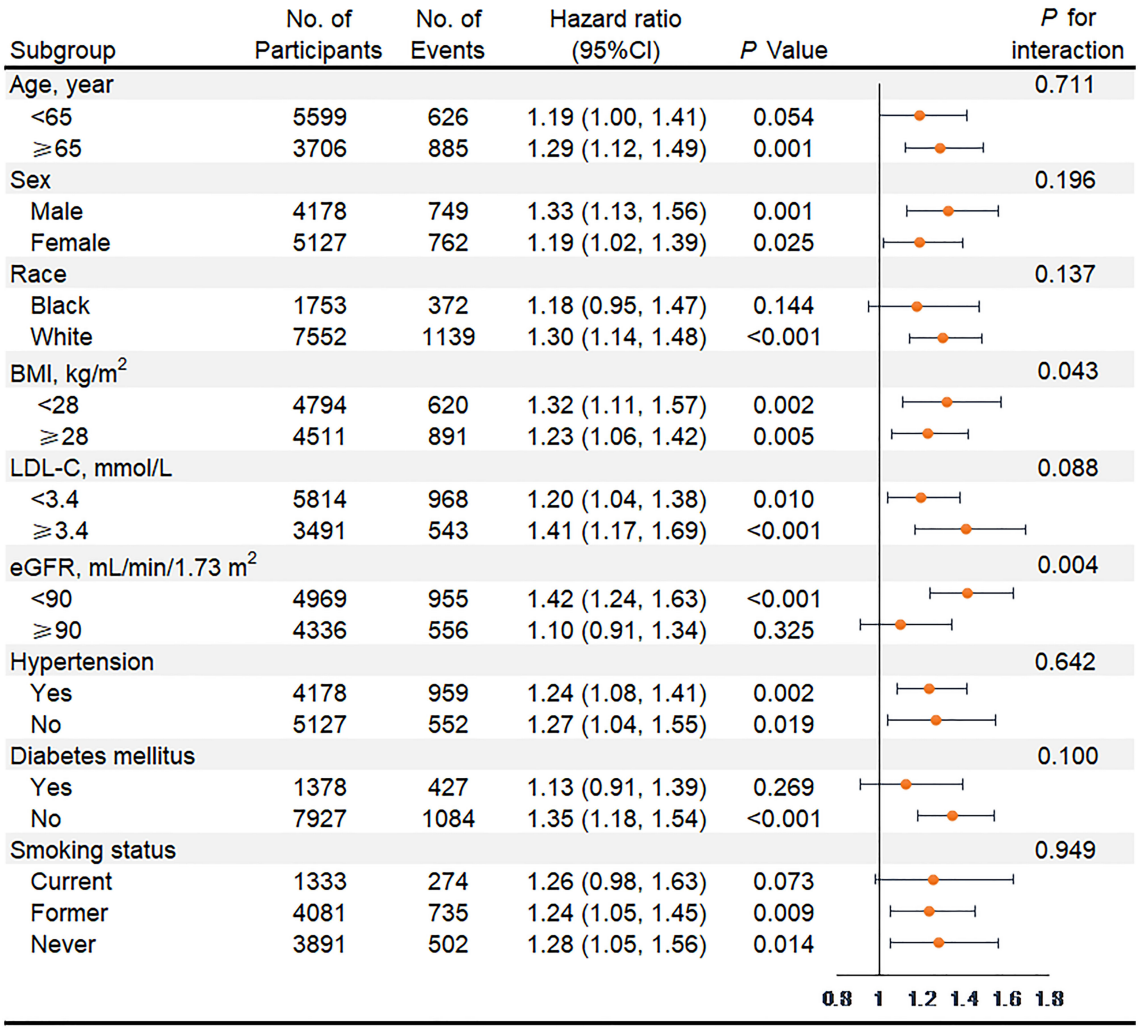
Association of the highest quartile of MAP variability group (VIM Q4) compared with the lower MAP variability group (VIM Q1 + Q2 + Q3) for heart failure in key subgroups. Hazard ratios and 95% CIs were obtained after individually removing the original variable from the Cox Model 3 that adjusted for age, sex, race, BMI, education level, smoking status, drinking status, total cholesterol, LDL-C, HDL-C, triglyceride, fasting glucose, eGFR, prevalent hypertension, diabetes mellitus, coronary heart disease, myocardial infarction, stroke, antihypertensive medicine, aspirin, statin, SBP, DBP at visit 4, and mean of MAP from visit 1 to 4. MAP, mean arterial pressure; VIM, variability independent of the mean; BMI, body mass index; LDL-C, low-density lipoprotein cholesterol; HDL-C, high-density lipoprotein cholesterol; eGFR, estimated glomerular filtration rate; SBP, systolic blood pressure; DBP, diastolic blood pressure.

In subgroup analyses of key variables, although the different risks of death in the subgroup of sex, race, previous diabetes, and smoking status and the different risks of HF in age, race, previous diabetes, and smoking status were found, interaction testing revealed no heterogeneity (Figures 3, 4). However, in the subgroup analysis of eGFR, the association between VIM of MAP and the risk of HF was just found in participants with eGFR <90 ml/min/1.73 m^2^ with a positive interaction (*P* = 0.004; Figure 4). Of note, the highest quartile of VIM was both associated with the risks of all-cause death and HF in participants with or without hypertension (all *P* < 0.05).

### Sensitivity Analyses

In sensitivity analyses for MAP variability measured by SD, CV, and ARV, the higher incident rate of death and HF were also found in the highest quartile of MAP variability compared with the lower MAP variability (Supplementary Figure 3). Consistent with the results of the primary analysis, the multivariable-adjusted COX model (Supplementary Tables 3, 4) and cubic spline curves (Supplementary Figure 4) both revealed that the highest quartile of MAP variability was associated with the higher risk of death and HF. In addition, we also conducted the sensitivity analysis in participants with normotension (*n* = 4,600), who were categorized into four groups by quartile value according to VIM of MAP. The characteristics are presented in Supplementary Table 5 with a lower heterogeneity. The results of the multivariable adjusted COX model also showed the higher risks of death and HF in the highest quartile of MAP variability (VIM Q4) (all-cause mortality: HR, 1.30; 95% CI, 1.09–1.55; HF: HR, 1.49; 95% CI, 1.12–1.98) (Supplementary Table 6). Similar results with the primary analysis were also found when the MAP_2_ was calculated by another formula (Supplementary Table 7).

## Discussion

In this analysis of data from the ARIC study, we found that higher long-term visit-to-visit MAP variability was associated with an increased risk of all-cause mortality and HF in participants with or without hypertension. The associations were independent of SBP, DBP, and mean MAP and other factors and were robust in the analysis of cubic spline curve and a number of sensitivity analyses, indicating that the BP variability assessed by MAP might be a potential risk factor, which might provide more accurate estimates for the risks of HF and death.

To the best of our knowledge, there is the first large prospective cohort study to date examining the association between long-term visit-to-visit MAP variability and HF and all-cause mortality. MAP, a main driving force for vital organ perfusion, was closely related to HF and all-cause mortality. In a Swedish prospective cohort study, Fedorowski et al. (27) found that the postural changes in MAP were associated with the incidence of the first hospitalization due to new-onset HF in 32,669 individuals over a follow-up of 24 years. Moreover, lower MAP was linked to increased all-cause mortality among 123 consecutive patients hospitalized for acute HF (18) and among 1,002 patients with cardiogenic shock (17). Our study reports the association of MAP variability with HF and death in a large cohort from the ARIC study. It adds to evidence that the MAP variability might also be a potential risk factor for HF and all-cause mortality. It might be informative, therefore, to restrain the extent of MAP variability for optimal BP management.

Prior studies have reported higher BP variability measured by SBP was associated with increased risks of all-cause mortality, CHD, stroke among 2,865,157 US veterans with and without hypertension (4) and among 16,758 participants aged 70 years and older without a history of CVD events from the Aspirin in Reducing Events in the Elderly (ASPREE) trial (14). The ASPREE trial also demonstrated a higher risk of HF events with higher SBP variability. In patients with type 2 diabetes from the Action to Control Cardiovascular Risk in Diabetes (ACCORD) trial and the Veterans Affairs Diabetes Trial (ADT), BP variability measured by SBP and DBP were both associated with increased risk of HF, even after adjusting for other risk factors and mean BP (9). The current study builds on these previous works to clarify that the MAP variability, similar to SBP or DBP variability, was also associated with the risks of HF and death. Our findings confirm and strengthen the importance of long-term BP variability for health-related outcomes from the perspective of MAP.

Although numerous studies indicated a higher risk of adverse cardiovascular events with higher BP variability, the reasons were unclear. Several mechanisms have been put forward to account for that. The increased BP variability might be associated with non-adherence to BP medications (28), disturbed baroreflex function leading to an exaggerated pressor response to emotional and physical stimuli (29), and changes in the elastic properties of blood vessels and aortic distensibility (30). These factors might account for the increased risk of adverse cardiovascular events. The previous study has shown that BP variability was correlated negatively with ankle–brachial index and positively with pulse wave velocity, suggesting a link between BP variability and impaired vascular function (31). Indeed, increased BP variability led to greater stress on blood vessels and endothelial dysfunction promoting early target-organ damage (32). Because both arterial stiffness and endothelial dysfunction have been implicated in the development of adverse cardiovascular events, they could also be plausible contributors to the development of HF. An additional possibility was that MAP variability might more directly decrease the myocardial perfusion. As MAP was a main driving force for vital organ perfusion (15), repeated transient reductions in MAP over time might put cardiac tissue at increased risk of relative hypoperfusion. More detailed studies elucidating the role of MAP variability, and the relevant mechanisms, in the development of death and HF are clearly needed to help refine our understanding of and guidance for optimal BP management.

The strengths of our study included its large sample size of almost 10 thousand individuals, followed with regular visits for a long period of time (16.8 years), and its representativeness of the community population. The main limitation was the analysis of observational data, which meant that we only reported associations and cannot make inferences about the causality of MAP variability in that we could not exclude the effect of residual measured or unmeasured confounders on our results. However, the impact of various confounding factors had been adjusted in our risk estimation models, and we performed separate subgroup analyses of key variables and several sensitivity analyses and found consistent results. Another possible confounding factor is that some degree of BP measurement error was unavoidable, even though all BP measurements were taken by trained staff according to standardized ARIC protocols and repeatability of measurements was high. In addition, it is challenging to evaluate the influence of environmental and behavioral factors and adherence to antihypertensive therapy on long-term MAP variability due to a lack of relevant data. Finally, the findings may lack generalizability to all regions and other racial and ethnic groups (e.g., Asian and Hispanic).

In conclusion, in individuals with or without hypertension, greater long-term visit-to-visit MAP variability was associated with a higher risk of all-cause mortality and HF, indicating that the BP variability assessed by MAP might be a potential risk factor for death and HF. Our study added to the current literature that linked BP variability with adverse cardiovascular outcomes from the perspective of MAP and might contribute to developing optimal BP management strategies.

## Data Availability Statement

Publicly available datasets were analyzed in this study. This data can be found at: https://sites.cscc.unc.edu/aric/.

## Ethics Statement

The studies involving human participants were reviewed and approved by the institutional review boards at all participating institutions of ARIC study (Forsyth County, North Carolina; Jackson, Mississippi; Washington County, Maryland; and Northwestern suburbs of Minneapolis, Minnesota). The patients/participants provided their written informed consent to participate in this study.

## Author Contributions

ML, XC, XZhu, and XL: had full access to all of the data in the study and take responsibility for the integrity of the data and the accuracy of the data analysis. XL and XZhu: concept and design, administrative, technical, or material support, and supervision. SZ, YL, ZX, XZho, YG, XS, HZ, XX, and LW: acquisition, analysis, or interpretation of data. ML and XC: drafting of the manuscript. SZ, YL, ZX, and XZho: statistical analysis. XL: obtained funding. All authors provided critical revision of the manuscript for important intellectual content.

## Conflict of Interest

The authors declare that the research was conducted in the absence of any commercial or financial relationships that could be construed as a potential conflict of interest.
