# Supplementary material for: Long-Term Visit-to-Visit Mean Arterial Pressure Variability and the Risk of Heart Failure and All-Cause Mortality
Source: Front Cardiovasc Med. 2021 Jun 4;8:665117. doi: 10.3389/fcvm.2021.665117 (PMC8211989; doi:10.3389/fcvm.2021.665117)
Supplement: Supplementary file 1 [file Data_Sheet_1.pdf]

Supplementary Material

1 Supplementary Figures and Tables

1.1 Supplementary Figures

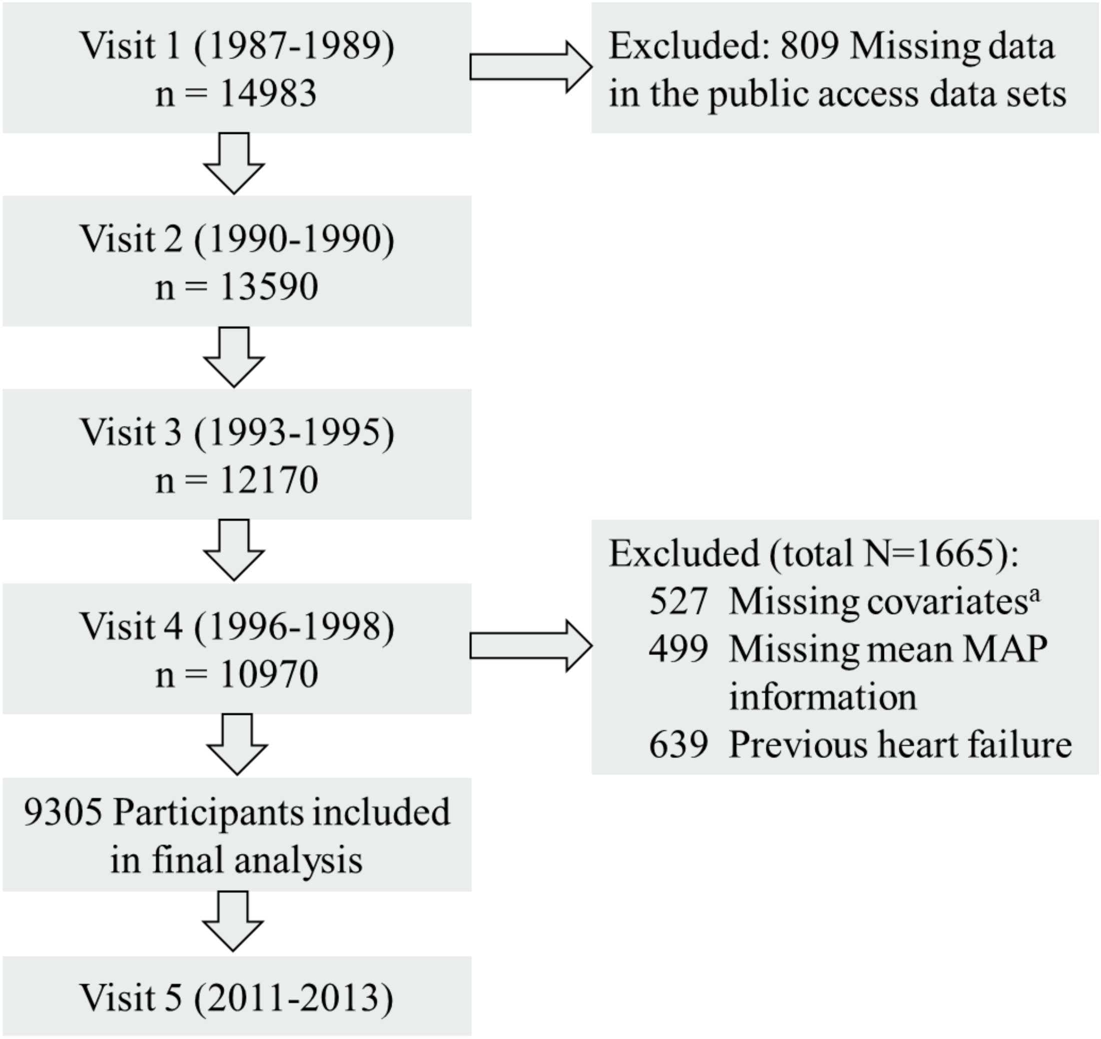

Supplementary Figure 1. Study flowchart with detailed study exclusion information.

MAP: mean arterial pressure; BMI: body mass index; LDL-C: low density lipoprotein cholesterol; [a] Missing covariates: 13 education level, 18 BMI, 199 LDL-C; 35 prevalent hypertension, 26 prevalent diabetes mellitus, 187 prevalent coronary heart disease, 12 prevalent stroke, 19 taking aspirin, 15 smoking status, 3drinking status.

$$SD = \sqrt{\frac{\sum_{i=1}^{4}(MAP_i - mean\ MAP)^2}{3}}$$

$$CV = \frac{SD}{mean\ MAP}$$

$$ARV = \frac{(|MAP_2 - MAP_1| + |MAP_3 - MAP_2| + |MAP_4 - MAP_3|)}{3}$$

$$VIM = SD \times (\frac{MAP}{mean\ MAP})^{\rho}$$

Supplementary Figure 2. The calculating formula of SD, CV, ARV and VIM.

MAP: mean arterial pressure; SD: standard deviation; CV: coefficient of variation; ARV: average real variability; VIM: variability independent of the mean; Mean MAP levels were calculated across 4 visits (visit 1, 2, 3 and 4) for each participant; $i = 1$ denotes visit 1 measurement; $i = 2$ to 4 denotes the measurements of visit 2 to visit 4; ρ is the regression coefficient on the basis of regressing the natural logarithm of SD on the natural logarithm of the mean MAP.

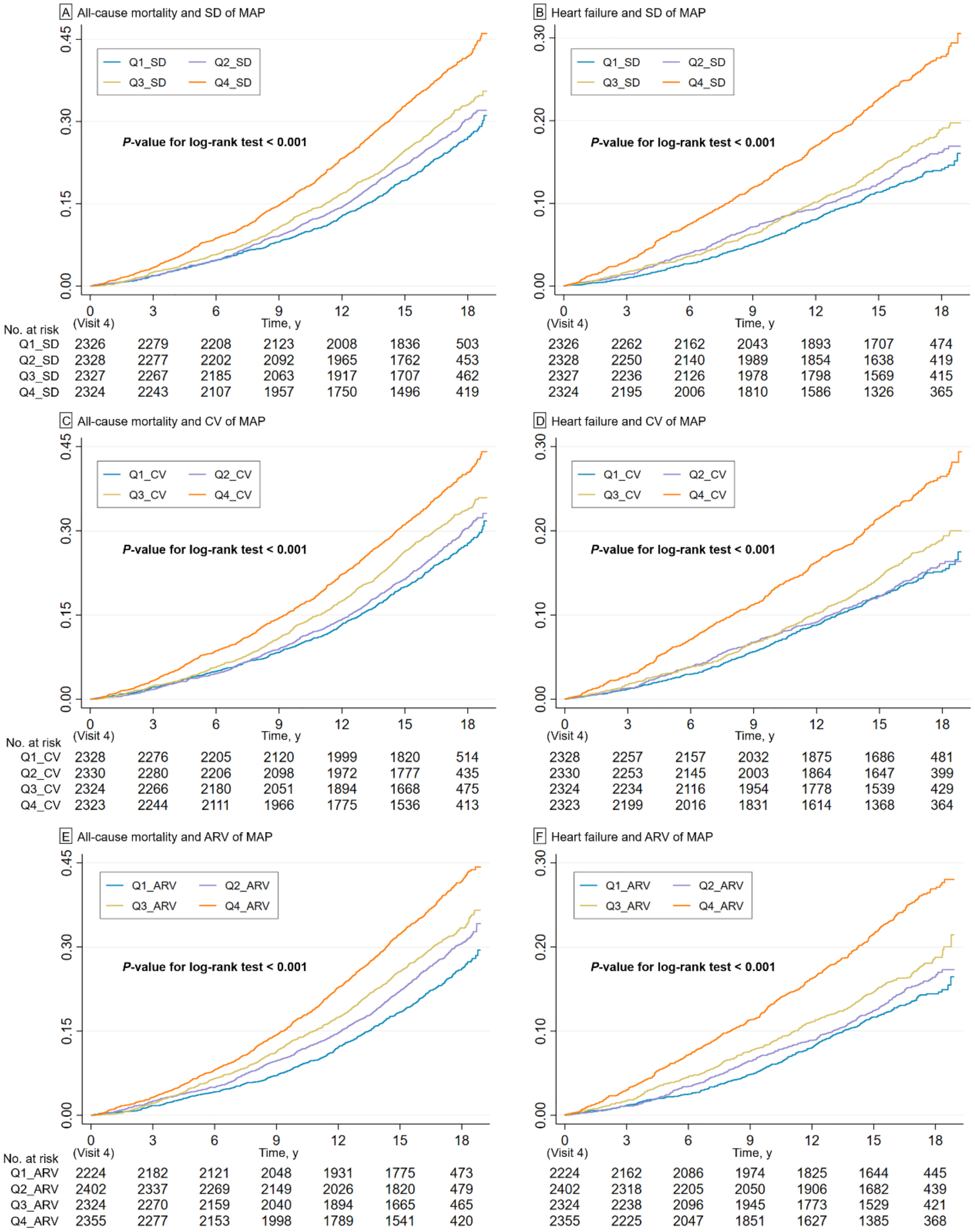

Supplementary Figure 3. Cumulative incidence estimates (Kaplan-Meier) for the all-cause mortality and heart failure in 4 groups by quartile value of MAP variability (SD, CV, ARV).

MAP: mean arterial pressure; VIM: variability independent of the mean.

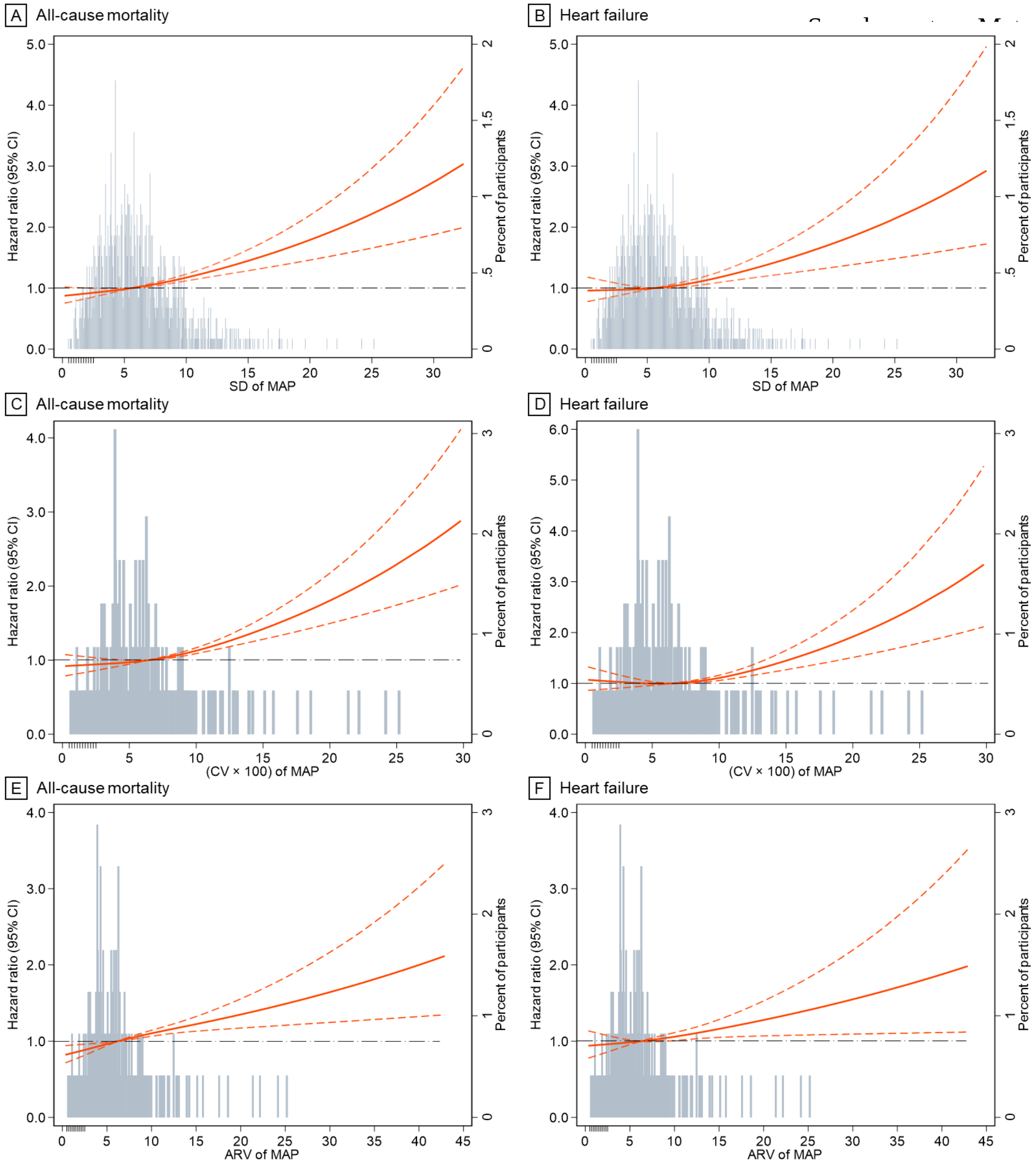

Supplementary Figure 4. Adjusted hazard ratios (95% CI) for the association of MAP variability measured by SD, CV×100 and ARV with incident all-cause mortality and heart failure.

(A) and (B): all-cause mortality and heart failure at MAP variability measured by SD; (C) and (D): all-cause mortality and heart failure at MAP variability measured by CV×100; (E) and (F): all-cause mortality and heart failure at MAP variability measured by ARV.

Hazard ratios (indicated by a red solid line) and 95% CIs (red dotted lines) are derived from Cox proportional hazard regression models adjusted for age, sex, race, BMI, education level, smoking status, drinking status, total cholesterol, LDL-C, HDL-C, triglyceride, fasting glucose, eGFR, prevalent hypertension, diabetes mellitus, coronary heart disease, myocardial infarction, and stroke, antihypertensive medicine, aspirin, statin, SBP, DBP at visit 4 and mean of MAP from visit 1 to visit 4. The SD, CV×100 and ARV was respectively centered at the sample median and modeled using a restricted cubic spline with knots at the 5th, 50th, and 95th percentiles. Black dotted line is the reference line as hazard ratio = 1. Histograms represent the frequency distribution of MAP variability (SD, CV×100 and ARV).

MAP: mean arterial pressure; SD: standard deviation; CV: coefficient of variation; ARV: average real variability; BMI: body mass index; LDL-C: low-density lipoprotein cholesterol; HDL-C: high-density lipoprotein cholesterol; eGFR: estimated glomerular filtration rate; SBP: systolic blood pressure; DBP: diastolic blood pressure.

1.2 Supplementary Tables

Supplementary Table 1. Correlations among variabilities of MAP (n = 9,305).

Variables	Mean MAP	SD_{MAP}	CV_{MAP}	ARV_{MAP}	VIM_{MAP}
Mean MAP	1	—	—	—	—
SD_{MAP}	0.326	1	—	—	—
CV_{MAP}	0.122	0.971	1	—	—
ARV_{MAP}	0.304	0.869	0.840	1	—
VIM_{MAP}	-0.052	0.906	0.980	0.780	1

Pearson's correlation coefficients are shown and all *P* values were less than 0.05. MAP variabilities were determined based upon 4 clinic blood pressure measurements from visit 1 to visit 4. MAP: mean arterial pressure; SD: standard deviation; CV: coefficient of variation; ARV: average real variability; VIM: variability independent of the mean.

Supplementary Table 2. Association of visit-to-visit MAP variability measured by VIM with the risk of CVD (n = 8,927).*

Variability	Model 1		Model 2		Model 3	
	Hazard Ratio (95% CI)	*P* Value	Hazard Ratio (95% CI)	*P* Value	Hazard Ratio (95% CI)	*P* Value
VIM Q1	1 (ref.)	—	1 (ref.)	—	1 (ref.)	—
VIM Q2	0.90 (0.77-1.05)	0.165	0.87 (0.74-1.01)	0.068	0.88 (0.76-1.03)	0.106
VIM Q3	0.98 (0.84-1.14)	0.816	0.91 (0.78-1.06)	0.227	0.93 (0.80-1.08)	0.346
VIM Q4	1.20 (1.0.4-1.40)	0.013	0.96 (0.83-1.12)	0.620	0.99 (0.85-1.15)	0.845
P for Trend	0.006		0.827		0.946	

* The 8,927 participants were included for this analysis about CVD outcome, excluding those who occurred CVD from visit 1 to visit 4 (n = 378).
Model 1: adjusted for age, sex, race at visit 4;
Model 2: adjusted for model 1 + education level, BMI, smoking status, drinking status, total cholesterol, HDL-C, LDL-C, triglyceride, fasting glucose, eGFR, prevalent hypertension, diabetes mellitus, coronary heart disease, myocardial infarction, and stroke, antihypertensive medicine, aspirin, statin at visit 4;
Model 3: adjusted for model 2 + SBP, DBP at visit 4 and Mean of MAP from visit 1 to visit 4.
MAP: mean arterial pressure; VIM: variability independent of the mean; CVD: cardiovascular disease; BMI: body mass index; HDL-C: high density lipoprotein cholesterol; LDL-C: low density lipoprotein cholesterol; eGFR: estimated glomerular filtration rate; SBP: systolic blood pressure; DBP: diastolic blood pressure.

Supplementary Table 3. Association of visit-to-visit MAP variability measured by SD, CV and ARV with all-cause mortality.

Variability	Model 1		Model 2		Model 3	
	Hazard Ratio (95% CI)	*P* Value	Hazard Ratio (95% CI)	*P* Value	Hazard Ratio (95% CI)	*P* Value
SD of MAP						
SD Q1	1 (ref.)	—	1 (ref.)	—	1 (ref.)	—
SD Q2	1.08 (0.96-1.20)	0.200	1.01 (0.90-1.13)	0.894	1.02 (0.91-1.14)	0.772
SD Q3	1.19 (1.07-1.33)	0.002	1.10 (0.98-1.22)	0.108	1.09 (0.98-1.22)	0.119
SD Q4	1.58 (1.42-1.75)	<0.001	1.27 (1.14-1.42)	<0.001	1.26 (1.13-1.41)	<0.001
CV of MAP						
CV Q1	1 (ref.)	—	1 (ref.)	—	1 (ref.)	—
CV Q2	1.05 (0.94-1.17)	0.390	0.99 (0.89-1.11)	0.847	1.00 (0.89-1.11)	0.943
CV Q3	1.21 (1.09-1.35)	0.001	1.11 (1.00-1.24)	0.054	1.12 (1.00-1.25)	0.045
CV Q4	1.50 (1.35-1.67)	<0.001	1.22 (1.10-1.36)	<0.001	1.22 (1.09-1.35)	<0.001
ARV of MAP						
ARV Q1	1 (ref.)	—	1 (ref.)	—	1 (ref.)	—
ARV Q2	1.17 (1.05-1.31)	0.005	1.09 (0.98-1.22)	0.128	1.09 (0.98-1.22)	0.124
ARV Q3	1.27 (1.13-1.41)	<0.001	1.15 (1.02-1.28)	0.018	1.14 (1.02-1.28)	0.021
ARV Q4	1.61 (1.45-1.79)	<0.001	1.31 (1.17-1.47)	<0.001	1.29 (1.15-1.44)	<0.001

Model 1: adjusted for age, sex, race at visit 4;

Model 2: adjusted for model 1 + education level, BMI, smoking status, drinking status, total cholesterol, HDL-C, LDL-C, triglyceride, fasting glucose, eGFR, prevalent hypertension, diabetes mellitus, coronary heart disease, myocardial infarction, and stroke, antihypertensive medicine, aspirin, statin at visit 4;

Model 3: adjusted for model 2 + SBP, DBP at Visit 4 and Mean of MAP from visit 1 to visit 4.

MAP: mean arterial pressure; SD: standard deviation; CV: coefficient of variation; ARV: average real variability; BMI: body mass index; HDL-C: high density lipoprotein cholesterol; LDL-C: low density lipoprotein cholesterol; eGFR: estimated glomerular filtration rate; SBP: systolic blood pressure; DBP: diastolic blood pressure.

Supplementary Table 4. Association of visit-to-visit MAP variability measured by SD, CV and ARV with heart failure events.

Variability	Model 1		Model 2		Model 3	
	Hazard Ratio (95% CI)	*P* Value	Hazard Ratio (95% CI)	*P* Value	Hazard Ratio (95% CI)	*P* Value
SD of MAP						
SD Q1	1 (ref.)	—	1 (ref.)	—	1 (ref.)	—
SD Q2	1.10 (0.94-1.29)	0.230	0.99 (0.85-1.16)	0.914	1.01 (0.86-1.18)	0.915
SD Q3	1.22 (1.04-1.42)	0.013	1.01 (0.87-1.19)	0.868	1.01 (0.86-1.18)	0.939
SD Q4	1.88 (1.62-2.17)	<0.001	1.30 (1.12-1.51)	0.001	1.25 (1.07-1.46)	0.005
CV of MAP						
CV Q1	1 (ref.)	—	1 (ref.)	—	1 (ref.)	—
CV Q2	0.98 (0.84-1.15)	0.834	0.90 (0.77-1.06)	0.198	0.92 (0.78-1.07)	0.278
CV Q3	1.14 (0.98-1.32)	0.096	1.00 (0.86-1.16)	0.987	1.01 (0.87-1.17)	0.918
CV Q4	1.68 (1.45-1.93)	<0.001	1.23 (1.06-1.42)	0.006	1.21 (1.05-1.40)	0.011
ARV of MAP						
ARV Q1	1 (ref.)	—	1 (ref.)	—	1 (ref.)	—
ARV Q2	1.09 (0.93-1.27)	0.300	0.97 (0.82-1.13)	0.658	0.96 (0.82-1.13)	0.663
ARV Q3	1.19 (1.02-1.39)	0.032	1.00 (0.86-1.17)	0.986	0.99 (0.85-1.16)	0.896
ARV Q4	1.75 (1.51-2.02)	<0.001	1.21 (1.04-1.41)	0.015	1.15 (0.99-1.34)	0.072

Model 1: adjusted for age, sex, race at visit 4;

Model 2: adjusted for model 1 + education level, BMI, smoking status, drinking status, total cholesterol, HDL-C, LDL-C, triglyceride, fasting glucose, eGFR, prevalent hypertension, diabetes mellitus, coronary heart disease, myocardial infarction, and stroke, antihypertensive medicine, aspirin, statin at visit 4;

Model 3: adjusted for model 2 + SBP, DBP at visit 4 and Mean of MAP from visit 1 to visit 4.

MAP: mean arterial pressure; SD: standard deviation; CV: coefficient of variation; ARV: average real variability; BMI: body mass index; HDL-C: high density lipoprotein cholesterol; LDL-C: low density lipoprotein cholesterol; eGFR: estimated glomerular filtration rate; SBP: systolic blood pressure; DBP: diastolic blood pressure.

Supplementary Table 5. Characteristics of each group in participants with normotension categorized by the VIM of MAP at visit 4.

Characteristics	Total	VIM Q1	VIM Q2	VIM Q3	VIM Q4	*P* value
No.	4600	1150	1150	1150	1150	
Age, years	61.8 (5.5)	61.7 (5.4)	61.8 (5.4)	61.9 (5.7)	62.0 (5.6)	0.495
Sex, No. (%)						<0.001
Men	2073 (45.1)	592 (51.5)	563 (49.0)	494 (43.0)	424 (36.9)	
Women	2527 (54.9)	558 (48.5)	587 (51.0)	656 (57.0)	726 (63.1)	
Race, No. (%)						0.007
Black	562 (12.2)	123 (10.7)	138 (12.0)	131 (11.4)	170 (14.8)	
White	4038 (87.8)	1027 (89.3)	1012 (88.0)	1019 (88.6)	980 (85.2)	
BMI, kg/m^2	27.4 (4.7)	27.7 (4.5)	27.6 (4.6)	27.3 (4.8)	27.0 (4.9)	0.001
Systolic BP, mm Hg	117.6 (11.8)	118.3 (11.0)	117.9 (11.2)	118.1 (12.1)	117.2 (12.9)	0.237
Diastolic BP, mm Hg	68.3 (8.5)	69.2 (7.3)	69.2 (7.7)	67.8 (8.4)	66.9 (10.1)	<0.001
MAP, mm Hg	84.7 (8.4)	85.6 (7.2)	85.4 (7.6)	84.2 (8.4)	83.7 (9.8)	<0.001
Total cholesterol, mmol/L	5.2 (0.9)	5.2 (0.9)	5.2 (0.9)	5.2 (0.9)	5.2 (1.0)	0.413
HDL-C, mmol/L	1.3 (0.4)	1.3 (0.4)	1.3 (0.4)	1.4 (0.5)	1.4 (0.4)	0.003
LDL-C, mmol/L	3.2 (0.9)	3.2 (0.9)	3.2 (0.8)	3.2 (0.9)	3.2 (0.9)	0.844
Triglyceride, mmol/L	1.5 (0.7)	1.5 (0.8)	1.5 (0.8)	1.5 (0.7)	1.5 (0.7)	0.140
Fasting Glucose, mmol/L	5.8 (1.7)	5.9 (1.7)	5.8 (1.7)	5.7 (1.5)	5.8 (1.8)	<0.001
eGFR, mL/min/1.73 m^2	87.8 (13.1)	87.1 (12.7)	88.0 (13.3)	87.6 (13.0)	88.5 (13.5)	0.069
Diabetes mellitus, No. (%)	400 (8.7)	117 (10.2)	87 (7.6)	89 (7.7)	107 (9.3)	0.512
Coronary heart disease, No. (%)	142 (3.1)	34 (3.0)	41 (3.6)	38 (3.3)	29 (2.5)	0.502
Myocardial infarction, No. (%)	120 (2.6)	31 (2.7)	27 (2.3)	34 (3.0)	28 (2.4)	0.795
Stroke, No. (%)	35 (0.8)	8 (0.7)	6 (0.5)	15 (1.3)	6 (0.5)	0.098
Education level, No. (%)						0.006
Basic or 0 y	622 (13.5)	135 (11.7)	161 (14.0)	162 (14.1)	164 (14.3)	
Intermediate	1981 (43.1)	492 (42.8)	468 (40.7)	502 (43.7)	519 (45.1)	
Advanced	1997 (43.4)	523 (45.5)	521 (45.3)	486 (42.3)	467 (40.6)	
Smoking, No. (%)						<0.001
Current smoker	732 (15.9)	133 (11.6)	169 (14.7)	170 (14.8)	260 (22.6)	
Former smoker	1962 (42.7)	518 (45.0)	507 (44.1)	500 (43.5)	437 (38.0)	
Never smoker	1906 (41.4)	499 (43.4)	474 (41.2)	480 (41.7)	453 (39.4)	
Drinking, No. (%)						0.001
Current drinker	2583 (56.2)	693 (60.3)	655 (57.0)	646 (56.2)	589 (51.2)	
Former drinker	1239 (26.9)	273 (184)	313 (27.2)	298 (25.9)	355 (30.9)	
Never drinker	778 (16.9)	184 (16.0)	182 (15.8)	206 (17.9)	206 (17.9)	
Aspirin, No. (%)	2313 (50.3)	560 (48.7)	587 (51.0)	602 (52.3)	564 (49.0)	0.252
Statin, No. (%)	301 (6.5)	65 (5.7)	76 (6.6)	84 (7.3)	76 (6.6)	0.458
VIM	5.7 (3.0)	2.5 (0.8)	4.4 (0.5)	6.1 (0.5)	9.6 (2.6)	<0.001

Continuous variables are presented as mean (SD), and categorical variables are presented as percentage. MAP: mean arterial pressure; VIM: variability independent of the mean; BMI: body mass index; BP: blood pressure; HDL-C: high density lipoprotein cholesterol; LDL-C: low density lipoprotein cholesterol; eGFR: estimated glomerular filtration rate.

Supplementary Table 6. Association of visit-to-visit MAP variability measured by VIM with all-cause mortality and heart failure events in participants with normotension.

Variability	Model 1		Model 2		Model 3	
	Hazard Ratio (95% CI)	*P* Value	Hazard Ratio (95% CI)	*P* Value	Hazard Ratio (95% CI)	*P* Value
All-Cause Mortality						
VIM Q1	1 (ref.)	—	1 (ref.)	—	1 (ref.)	—
VIM Q2	1.15 (0.96-1.37)	0.122	1.11 (0.93-1.32)	0.256	1.12 (0.94-1.33)	0.218
VIM Q3	1.20 (1.01-1.42)	0.043	1.12 (0.94-1.34)	0.203	1.13 (0.95-1.35)	0.163
VIM Q4	1.49 (1.25-1.76)	<0.001	1.29 (1.09-1.54)	0.003	1.30 (1.09-1.55)	0.003
Heart Failure						
VIM Q1	1 (ref.)	—	1 (ref.)	—	1 (ref.)	—
VIM Q2	1.07 (0.80-1.43)	0.669	1.05 (0.78-1.41)	0.761	1.08 (0.80-1.45)	0.626
VIM Q3	1.36 (1.03-1.79)	0.031	1.35 (1.02-1.79)	0.036	1.41 (1.07-1.87)	0.016
VIM Q4	1.52 (1.15-2.00)	0.003	1.41 (1.07-1.87)	0.015	1.49 (1.12-1.98)	0.006

Model 1: adjusted for age, sex, race at visit 4;

Model 2: adjusted for model 1 + education level, BMI, smoking status, drinking status, total cholesterol, HDL-C, LDL-C, triglyceride, fasting glucose, eGFR, prevalent diabetes mellitus, coronary heart disease, myocardial infarction, and stroke, aspirin, statin at visit 4;

Model 3: adjusted for model 2 + SBP, DBP at visit 4 and Mean of MAP from visit 1 to visit 4.

MAP: mean arterial pressure; VIM: variability independent of the mean; BMI: body mass index; HDL-C: high density lipoprotein cholesterol; LDL-C: low density lipoprotein cholesterol; eGFR: estimated glomerular filtration rate; SBP: systolic blood pressure; DBP: diastolic blood pressure.

Supplementary Table 7. Association of visit-to-visit MAP_2 variability measured by VIM with all-cause mortality and heart failure events.

Variability	Model 1		Model 2		Model 3	
	Hazard Ratio (95% CI)	*P* Value	Hazard Ratio (95% CI)	*P* Value	Hazard Ratio (95% CI)	*P* Value
All-Cause Mortality						
VIM Q1	1 (ref.)	—	1 (ref.)	—	1 (ref.)	—
VIM Q2	1.09 (0.98-1.22)	0.106	1.05 (0.94-1.17)	0.355	1.06 (0.95-1.18)	0.278
VIM Q3	1.13 (1.02-1.26)	0.022	1.06 (0.96-1.19)	0.264	1.08 (0.97-1.20)	0.188
VIM Q4	1.51 (1.36-1.67)	<0.001	1.25 (1.12-1.39)	<0.001	1.26 (1.14-1.40)	<0.001
P for Trend	<0.001		<0.001		<0.001	
Heart Failure						
VIM Q1	1 (ref.)	—	1 (ref.)	—	1 (ref.)	—
VIM Q2	0.92 (0.79-1.08)	0.314	0.87 (0.75-1.02)	0.084	0.88 (0.76-1.03)	0.114
VIM Q3	1.03 (0.89-1.19)	0.721	0.95 (0.82-1.10)	0.487	0.97 (0.83-1.13)	0.679
VIM Q4	1.55 (1.35-1.78)	<0.001	1.20 (1.04-1.38)	0.013	1.22 (1.06-1.40)	0.007
P for Trend	<0.001		0.003		0.001	

Model 1: adjusted for age, sex, race at visit 4;

Model 2: adjusted for model 1 + education level, BMI, smoking status, drinking status, total cholesterol, HDL-C, LDL-C, triglyceride, fasting glucose, eGFR, prevalent hypertension, diabetes mellitus, coronary heart disease, myocardial infarction, and stroke, antihypertensive medicine, aspirin, statin at visit 4;

Model 3: adjusted for model 2 + SBP, DBP at visit 4 and Mean of MAP_2 from visit 1 to visit 4.

The MAP_2 calculated by another formula: $MAP_2 = DBP + 0.412 \times (SBP-DBP)$.

MAP: mean arterial pressure; VIM: variability independent of the mean; BMI: body mass index; HDL-C: high density lipoprotein cholesterol; LDL-C: low density lipoprotein cholesterol; eGFR: estimated glomerular filtration rate; SBP: systolic blood pressure; DBP: diastolic blood pressure.
